# Two Carbohydrate-Based Natural Extracts Stimulate *in vitro* Pollen Germination and Pollen Tube Growth of Tomato Under Cold Temperatures

**DOI:** 10.3389/fpls.2021.552515

**Published:** 2021-10-07

**Authors:** Ferdousse Laggoun, Nusrat Ali, Sabine Tourneur, Grégoire Prudent, Bruno Gügi, Marie-Christine Kiefer-Meyer, Alain Mareck, Florence Cruz, Jean-Claude Yvin, Eric Nguema-Ona, Jean-Claude Mollet, Frank Jamois, Arnaud Lehner

**Affiliations:** ^1^UNIROUEN, Normandie Université, Laboratoire de Glycobiologie et Matrice Extracellulaire Végétale, SFR NORVEGE FED 4277, Carnot I2C, IRIB, Rouen, France; ^2^Sanofi Pasteur, Val-de-Reuil, France; ^3^Centre Mondial de l’Innovation, Laboratoire Nutrition Végétale, Groupe Roullier, Saint-Malo, France; ^4^Laboratoire de Biologie et Pathologie Végétales, Université de Nantes, Université Bretagne Loire, Nantes, France

**Keywords:** carbohydrate, pollen germination and tube growth, ROS, tomato, callose, pectin methylesterase, cold temperature

## Abstract

To date, it is widely accepted by the scientific community that many agricultural regions will experience more extreme temperature fluctuations. These stresses will undoubtedly impact crop production, particularly fruit and seed yields. In fact, pollination is considered as one of the most temperature-sensitive phases of plant development and until now, except for the time-consuming and costly processes of genetic breeding, there is no immediate alternative to address this issue. In this work, we used a multidisciplinary approach using physiological, biochemical, and molecular techniques for studying the effects of two carbohydrate-based natural activators on *in vitro* tomato pollen germination and pollen tube growth cultured *in vitro* under cold conditions. Under mild and strong cold temperatures, these two carbohydrate-based compounds significantly enhanced pollen germination and pollen tube growth. The two biostimulants did not induce significant changes in the classical molecular markers implicated in pollen tube growth. Neither the number of callose plugs nor the *CALLOSE SYNTHASE* genes expression were significantly different between the control and the biostimulated pollen tubes when pollens were cultivated under cold conditions. PECTIN METHYLESTERASE (PME) activities were also similar but a basic PME isoform was not produced or inactive in pollen grown at 8°C. Nevertheless, NADPH oxidase (*RBOH*) gene expression was correlated with a higher number of viable pollen tubes in biostimulated pollen tubes compared to the control. Our results showed that the two carbohydrate-based products were able to reduce *in vitro* the effect of cold temperatures on tomato pollen tube growth and at least for one of them to modulate reactive oxygen species production.

## Introduction

Temperature and hydric stresses are the main limiting factors for crop production (Lamaoui et al., 2018). Many reports from the IPCC (Intergovernmental Panel on Climate Change) have pointed out that most agricultural regions will experience more extreme environmental fluctuations including severe variation of temperature and/or water availability (IPCC, 2014; Hoegh-Guldberg et al., 2018). As drought, cold temperature stresses can be detrimental to all phases of plant development (Yadav, 2010; Hussain et al., 2018) including the critical steps of gametophyte development and growth in flowering plants (Zinn et al., 2010; Giorno et al., 2013; Hatfield and Prueger, 2015; Mesihovic et al., 2016). This is an important challenge as the majority of our food supply is dependent on sexual reproduction. As recently reviewed by Pacini and Dolferus (2019) and demonstrated by several research papers, the process of sexual reproduction is very sensitive to low temperatures and impacts fruit and seed sets (Thakur et al., 2010; Barlow et al., 2015; Hatfield and Prueger, 2015; Keller et al., 2018). As reviewed by Zinn et al. (2010), cold temperature stresses have several major effects on reproductive tissues such as the timing of flowering, abnormal formation of parental organs, asynchrony of male and female maturation, stigma receptivity, reduction in pollen viability and germination and decrease in pollen tube growth both *in vivo* and *in vitro* (Zamir et al., 1981; Kakani et al., 2002; Boavida and McCormick, 2007). This detrimental effect or cold temperature stress on plant reproduction has been shown in different grain crops such as rice (Imin et al., 2004), wheat (Chakrabarti et al., 2011; Barlow et al., 2015), chickpea (Clarke and Siddique, 2004), and soybean (Ohnishi et al., 2010). Similarly to what was shown during drought, redox-regulatory network is modified in plant during cold stress acclimation, and the response of plants’ low temperature stress is the production and accumulation of reactive oxygen species (ROS; Hussain et al., 2018). Among ROS, anion superoxide (O_2_^•−^) is produced in part by NADPH oxidases (called respiratory burst oxidase homologs, Rbohs), hydrogen peroxide (H_2_O_2_) produced from O_2_^•−^ by superoxide dismutases, and/or the hydroxyl radical (OH^•^) produced by the non-enzymatic Fenton reaction (Mittler, 2017; Dreyer and Dietz, 2018). Excessive ROS production can cause oxidative damages to proteins, DNA, and lipids. They can alter cell wall stiffening in the presence of peroxidases by cross-linking glycoproteins or local polysaccharide cleavage with OH^•^, but they also could serve as signaling molecules (Suzuki and Mittler, 2006; Le Gall et al., 2015; Tenhaken, 2015; Airianah et al., 2016; Mittler, 2017; Novaković et al., 2018). In tomato, recent studies pointed out the role of ROS in vegetative tissues during cold stresses (Liu et al., 2020; Zhang et al., 2020). In reproductive tissues, ROS and most likely OH^•^ were involved in reproductive tissues, and ROS and most likely OH^•^ were involved in the pollen tube rupture to release the sperm cells in the ovule (Duan et al., 2014; Ge et al., 2019). Interestingly, in the freeze, cold or heat-tolerant plants, an over-accumulation of ROS scavenging enzymes such as APX (ascorbate peroxidase) and catalase (Saruyama and Tanida, 1995; Suzuki and Mittler, 2006) has been reported.

In tip-growing cells, such as root hairs and pollen tubes, the important role of ROS has been highlighted (Foreman et al., 2003; Boisson-Dernier et al., 2013). In pollen, ROS were shown to be involved in signaling for pollen grain germination and pollen tube growth (Potocký et al., 2007; Speranza et al., 2012; Muhlemann et al., 2018). The use of diphenylene iodonium (DPI), a NADPH oxidase inhibitor, reduced pollen tube growth (Potocký et al., 2007) and eventually led to pollen tubes burst (Speranza et al., 2012). In *Arabidopsis thaliana*, pollen-expressed NADPH oxidases, RbohH and RbohJ, were responsible for the oscillation of H_2_O_2_ at the tip of growing pollen tubes and the double *rbohH::rbohJ* mutant displayed almost 100% of bursting pollen tubes (Boisson-Dernier et al., 2013) revealing an important function of ROS as signaling molecules in tip-growing plant cells (Kaya et al., 2014). Interestingly, as observed with ROS, a tip-focused gradient of cytosolic calcium is also necessary for pollen tube growth (Konrad et al., 2011), and it has been suggested that calcium influx activates RBOH leading to the production of ROS in the cell wall which in turn could activate calcium influx (Novaković et al., 2018).

The fast growth of pollen tubes is promoted by a massive secretion of membrane and cell wall materials at the tip. Among them, pectins like the homogalacturonan (HG) motif are deposited under a highly methylesterified form and are demethylesterified in the sub-apical region by pectin methylesterases (PMEs) allowing the stiffening of the cell wall by interaction with calcium of the negative charges of galacturonic acids, the main monosaccharide of pectins (Dardelle et al., 2010; Chebli et al., 2012; Mollet et al., 2013; Dehors et al., 2019). The importance of PMEs was highlighted in *Arabidopsis* by the study of pollen-specific *pme* mutants. PME48 was shown to be involved in pollen germination (Leroux et al., 2015) and VANGUARD and AtPPME1 in pollen tube growth (Jiang et al., 2005; Tian et al., 2006). Additionally, in order to maintain the vegetative cell, carrying the two sperm cells, in the apical region of the pollen tube, periodic callose plugs are synthesized *via* plasma membrane-localized callose synthases (CalS)/Glucan synthase-like (GSL; Chen and Kim, 2009; Abercrombie et al., 2011; Zaveska Drabkova and Honys, 2017; Tucker et al., 2018; Dehors et al., 2019). In *A. thaliana* and tomato, a relationship was shown between the number of callose plugs, the position of the first callose plug and the pollen tube length (Qin et al., 2012).

Today, addressing the issue of climate change threat on plant reproduction requires a range of avoidance and corrective/preventive solutions. One of the solutions to overcome this problem is to select species, ecotypes and/or cultivars that can respond to temperature stress as it was investigated in rice (Prasad et al., 2006), tomato (Zamir et al., 1982), chickpea (Clarke and Siddique, 2004), bean (Porch and Jahn, 2001), or groundnut (Kakani et al., 2002).

Another solution to respond to the detrimental effects of thermal stresses is to find bioactive compounds that can stimulate pollen germination and pollen tube growth under stress conditions. Interestingly, cold and drought stresses shared common effects and cellular responses in plants (Beck et al., 2007; Hussain et al., 2018). Several studies showed the beneficial effect of the application of different compounds upon abiotic stresses. As reviewed by Hussain et al. (2018), plant growth regulators, mineral nutrients, and compatible solutes such as proline and soluble sugars were shown to improve plant responses to chilling and drought stresses. In *Prunus dulcis*, low exogenous supply of polyamines (putrescine, spermidine, or spermine) was shown to stimulate pollen tube growth at 10°C compared to the untreated conditions, while the same concentrations of compounds were detrimental at 25°C (Sorkheh et al., 2011).

In this study, we investigated the impact of two carbohydrate-based natural products (named P1 and P2) which were selected from a previous screen consisting of an in-house natural extract library, on their abilities to stimulate *in vitro Solanum lycopersicum* (tomato) pollen germination and pollen tube growth under thermic stress (4, 8, 13, 22, and 28°C). No pollen germination was observed at 4°C in any condition; however, both P1 and P2 were able to stimulate *in vitro* pollen germination and pollen tube growth at low temperatures (8 and 13°C) compared to untreated pollen. Therefore, to understand the stimulatory effects of these two carbohydrate-based activators, we investigated molecular markers of pollen tube growth such as gene expression of several *CalS* and *Rboh* genes, callose plug number, and PME activity.

## Materials and Methods

### Plant Growth Conditions

*Solanum lycopersicum* var. *cerasiforme* “West Virginia 106” (WVa106) seeds (a gift of Dr. Pierre Baldet, INRAE, Bordeaux, France) were sown 1cm under the surface of sterilized soil and cultured in a growth chamber. Plants were grown in optimal conditions with a photoperiod of a 16-h light/8-h dark cycle at 25 and 22°C during the light and dark period, respectively. Relative humidity was maintained at 60%, and plants were watered every 2 d. At flowering, flowers were harvested before each experiment to collect pollens as described below.

### Pollen Tube Culture and Treatments

Pollen grains were collected from freshly dehisced anthers, and the stamens of five flowers were submerged in 5ml of BK medium [1.62mM H_3_BO_3_, 1.25mM Ca(NO_3_)_2_, 4H_2_O, 2.97mM KNO_3_ and 1.65mM MgSO_4_, 7H_2_O] containing 10% (w/v) sucrose (Brewbaker and Kwak, 1963). Pollen grains were suspended in the BK medium by vortex, and the stamens were removed with tweezers. Tomato pollen tubes were grown in 24-well plates (ThermoFisher®), in the dark, without agitation at five different temperatures: 4, 8, 13, 22, and 28°C. Observations were performed after 2, 4, and 6h of growth. Pollen was considered germinated if the length of the pollen tube exceeded the diameter of the pollen grain.

The treatments consisted of two natural carbohydrate-based extracts (P1 and P2) selected from a previous screen of an in-house natural extract library. P1 and P2 are aqueous solution made of water containing 2.5% (w/v) of the active ingredient 1 and 4% (w/v) of the active ingredient 2, respectively. P1 and P2 were diluted with milli-Q water to reach a final concentration of active ingredient of 300μg.ml^−1^. Dose–response tests were performed at final concentrations (active ingredient) of 1, 2, 5, 10, 75, 150, and 300μg.ml^−1^ in the BK medium. Milli-Q water was used as a negative control (Supplementary Table S1). Further experiments were all carried out at the final concentration of 2μg.ml^−1^. The mix of P1 and P2 (Supplementary Table S2) corresponds to 2μg.ml^−1^ of active ingredient 1 and 2μg.ml^−1^ of active ingredient 2.

### Monosaccharide Composition

Products and active ingredients were analyzed by gas chromatography coupled to a flame ionization detector (GC-FID) spiking 50μl of 2-mM inositol as an internal standard as previously described (Dardelle et al., 2010). Briefly, each sample (0.5mg) was subjected to hydrolysis with 2M trifluoroacetic acid (TFA) for 2h at 110°C. TFA was washed twice with a 50% (v/v) *iso*-propanol/water solution. The released monosaccharides were converted to their *O*-methyl glycosides by incubation in 1M methanol/HCl at 80°C overnight. The methyl glycosides were then converted into their trimethylsilyl derivatives by heating the samples for 20min at 110°C in hexamethyldisilazane/trimethylchlorosilane/pyridine (3/1/9). After evaporation of the reagent, the samples were suspended in cyclohexane before being injected on a CP-Sil 5 CB column (Agilent Technologies). Chromatographic data were integrated with GC Star Workstation software (Varian). A temperature program (3min at 40°C; up to 160°C at 15°C min^−1^; up to 220°C at 1.5°C min^−1^; up to 280°C at 20°C min^−1^; 3min at 280°C) optimized for the separation of the most common cell wall authentic monosaccharides such as arabinose (Ara), rhamnose (Rha), fucose (Fuc), xylose (Xyl), glucuronic acid (GlcA), mannose (Man), mannitol, galactose (Gal), galacturonic acid (GalA), and glucose (Glc).

### Cytochemical Staining of Callose

Pollen tubes were fixed after a duration of culture corresponding to 4 times the D (duration necessary to get 50% of the highest germination rate of the control for each temperature; Table 1). The fixation medium composed of 100mM PIPES, 4mM MgSO_4_, 7 H_2_O, 4mM EGTA, 10% (w/v) sucrose, and 5% (v/v) of paraformaldehyde; pH 7.5 was added to the BK medium and incubated overnight at 4°C. Pollen tubes were centrifuged at 4,000*g* for 7min. The pellet was resuspended in 250μl of fresh BK. At 22 and 13°C, 0.3% decolorized aniline blue (DAB) prepared in 100-mM phosphate buffer pH 12 (Johnson-Brousseau and McCormick, 2004) was added to the medium at a final concentration of 0.05%. At 8°C, a double staining with DAB and calcofluor white was used at final concentrations of 0.05 and 0.1%, respectively. Observations were performed after 2h of incubation in the dark at room temperature.

**Table 1:** Values (h) of the 4*D at 8, 13, and 22°C for the control or pollen grains treated with 2μg.ml^−1^ of P1 or P2.

Treatment	Duration			4*D_8°_C	4*D_13°_C	4*D_22°_C
Control	18h40	7h	3h30
P1	12h20	5h20	3h20
P2	15h	4h	3h

D represents the duration necessary to reach 50% of the highest germination rate of the control for a given temperature.

### Microscope Observation and Image Acquisition

An inverted microscope Leica DMI 6000B equipped with the Leica DFC 450 camera was used to observe under bright-field the time course of pollen germination and pollen tube growth. Callose deposition was observed under epi-fluorescence using a filter with absorption 405nm and emission 523nm.

The program ImageJ (Abramoff et al., 2004) was used to measure pollen tube germination rate, pollen tube length, callose plug numbers, and diameters of the colored halos in the gel diffusion assay.

### RNA Extraction and Gene Expression Analysis

The RNA was extracted from the pollen culture of two tomato flowers in 2ml of BK germination medium for each treatment (control, P1 and P2) at 8, 13, and 22°C. Samples were collected at D_8°_C, D_13°_C, and D_22°_C (Table 2). At first, the germination medium was removed from pollen culture after centrifugation and replaced with 1ml of NucleoZOL (Macherey-Nagel, Düren, Germany), following which the pollen tubes were split by flow-reflux and vortexed. Total RNA extraction was carried out using Nucleospin® RNA set for NucleoZOL kit following the manufacturer’s protocol (Macherey-Nagel, Düren, Germany) with slight modifications. The quality of extracted RNA was checked in 4200 Tapestation (Agilent Technologies, CA, USA), followed by DNase treatment (Turbo DNA-free® kit) and cDNA synthesis from 1μg RNA, using iScript™ gDNA clear cDNA synthesis kit (Bio-Rad, CA, USA). The diluted cDNA samples were used to study gene expression by quantitative real-time PCR (CFX384 Touch™, Bio-Rad, CA, USA) in a total volume of 10μl using SsoAdvanced Universal SYBR Green Supermix (Bio-Rad, CA, USA). All the qPCR reactions were performed in technical triplicates using independent cDNA reactions for each of the six biological replicates and 300nM of gene-specific primer pairs. The thermal cycling protocol included a single polymerase activation step at 98°C for 3min followed by 40 amplification cycles, a final extension step at 72°C for 5min as well as melt-curve analysis. Each amplification cycle comprised a denaturation step at 98°C for 15s, a primer annealing step for 30s and a brief extension at 72°C for 15s. For each primer pair, primer efficiency was determined by performing standard curve analysis using serial dilutions. All qPCR expression data were acquired and analyzed using CFX Maestro Software Version 1.1 (Bio-Rad, CA, USA).

**Table 2:** D values (h) at 8, 13, and 22°C for the control and treated pollen grains with 2μg.ml^−1^ of P1 or P2.

Treatment	Duration			D_8°_C	D_13°_C	D_22°_C
Control	4h40	1h45	0h53
P1	3h05	1h20	0h50
P2	3h45	1h	0h45

D represents the duration required to reach 50% of the highest germination rate of the control for a given temperature.

The genes of interest for qRT-PCR analysis were identified in the tomato genome by similarity to their counterparts in the genome of *A. thaliana*. The tomato *CalS* candidate genes were selected using the protein sequence from the *Arabidopsis thaliana CalS5* gene (At2g13680) and the pollen-expressed *Rboh* genes (*RbohH* At5g60010 and *RbohJ* At3g45810) in a TBLASTN analysis (Altschul et al., 1997). The search was carried out in the NCBI Transcript references Sequences (refseq_rna) restricted to *Solanum lycopersicum* to retrieve transcripts corresponding to potential *CalS* and pollen-expressed *Rboh* cDNA sequences from tomato.

Nine putative tomato CalsS cDNA sequences (XM_019211256.2, XM_004228545.4, XM_026029202.1, XM_026031249.1, XM_010325327.3, XM_004236267.4, XM_010318449.3, XM_10318448.3 and XM_004232827.4) were selected in the NCBI database. They correspond, respectively, to the Sequence Id Solyc11g005980.2, Solyc11g005985.1, Solyc01g006370.3, Solyc01g073750.3, Solyc07g056260.3, Solyc03g111570.3 and Solyc02g078230.2 in the “Tomato Genome cDNA (ITAG release 3.20)” BLAST dataset (https://solgenomics.net/; Fernandez-Pozo et al., 2015). Two cDNA sequences coding potential Rboh were identified in GenBank (XM_019214404.2 and XM_004251404.4), corresponding, respectively, to the Sequence Id Solyc06g075570.1 and Solyc11g072800.1. The expression of the genes of interest was normalized against four reference genes: EXP, LZ, EF1α, and CK2A (Solyc07g025390.2, Solyc05g055770.2, Solyc06g005060.2, Solyc02g064700.2). Specific primers for all candidate and reference genes are listed in Supplementary Table S3. The primer search was performed using the Primer-BLAST program at the NCBI.^1^

### Protein Extraction and Protein Assay

Pollen from 10 flowers was incubated as described in “*Pollen tube culture and treatments*” for a duration corresponding to 4*D (Table 1) and frozen at −20°C until used. Tubes were thawed and pollen tubes were collected by centrifugation at 10000*g* for 3min. The supernatant was discarded, and the cell wall proteins from the pellet were extracted by grinding pollen tubes with a Fastprep (MP Biomedicals) in 200μl of 50mM Na_2_HPO_4_ 7H_2_O, 20mM citric acid, 1M NaCl, 0.01% (w/v) Tween 20, and 2mM PMSF, pH 7. The extract was incubated under agitation (200rpm) for 1h at 4°C. Cellular fragments were removed by centrifugation at 14000*g* for 30min. The crude protein extract was concentrated by ultrafiltration on Pierce Concentrator 10K MWCO, 0.5ml (Thermo Fisher) in milli-Q water. Proteins were quantified by the micromethod of Bradford (1976), with the Bio-Rad kit, and bovine serum albumin was used as a standard.

### PME Gel Diffusion Assay

Total PME activity was quantified by a gel diffusion assay (Downie et al., 1998) with some modifications. A solution containing 0.1% (w/v) citrus pectin with a degree of methylesterification (DM) of 85% (Sigma), 10mM citric acid, 20mM Na_2_HPO_4_ 7H_2_O, and 1% (w/v) agarose at pH 7 was transferred to Petri dishes (12cm x12 cm) and allowed to solidify. Wells were made in the gel with a 4-mm-diameter cork borer. A volume corresponding to 20μg of proteins was loaded in each well. The standard curve was obtained using a commercial orange PME (Sigma, Cat. No. P5400) ranging from 0.3 to 5U. Petri dishes were incubated for 16h at 37°C. After incubation, the gels were rinsed with milli-Q water and stained with 0.01% (w/v) ruthenium red (Sigma) for 1h. The dye was then poured off and the gels were rinsed with milli-Q water and scanned.

### IEF and Zymogram

Isoelectric focusing (IEF) electrophoresis and zymography were performed as described by Paynel et al. (2014). Briefly, IEF of native proteins was performed on SERVALYT™ PRECOTES™ gels with a 3 to 10 pH range (SERVA). Samples with identical PME activities (0.05U) were loaded in each well. Three μL of IEF marker liquid mix (SERVA) were loaded. After IEF, the track with pI markers was cut and stained with coomassie blue. The rest of the gel was washed for 15 to 30min in 20mM Tris-HCl, 5mM EDTA at pH 8.5. Activity of PME was then monitored in liquid (zymogram) by using a solution of 1% (w/v) citrus pectin with a DM of 85% (Sigma-Aldrich) dissolved in 20mM Tris-HCl, 5mM EDTA, 160mM NaCl, pH 7.5. The gel was incubated for 2h at 28°C, and the demethylated pectins resulting from PME activities were precipitated with 0.1M malic acid during 1h and washed with milli-Q water. The gel was finally stained overnight with 0.02% (w/v) ruthenium red and scanned.

### Statistical Analyses

For all experiments, data correspond to the mean of six biological replicates performed at different dates. Significant differences between the control and the treatment were determined by one-way ANOVA followed by Dunnett’s multiple comparison test. Data are marked by different letters when significantly different with respect to the control conditions (*p*-value<0.05). For germination, it represents between 1,000 and 2000 pollen grains counted for each replicates and time points. For pollen tube length, the number of measure ranged from 80 to 120 for each replicate.

## Results

Among the five temperatures tested, no germination was observed in any treatments after 16h of growth at 4°C. Consequently, in the following, only the data for 8, 13, 22 and 28°C are presented. The sugar composition of the bioactive-based carbohydrate extracts was analyzed using gas chromatography, and the effects of the products on the ability of the pollen grain to germinate and to produce healthy pollen tubes were analyzed.

### Monosaccharide Composition of the Two Carbohydrate-Based Natural Activators

**Figure 1:**
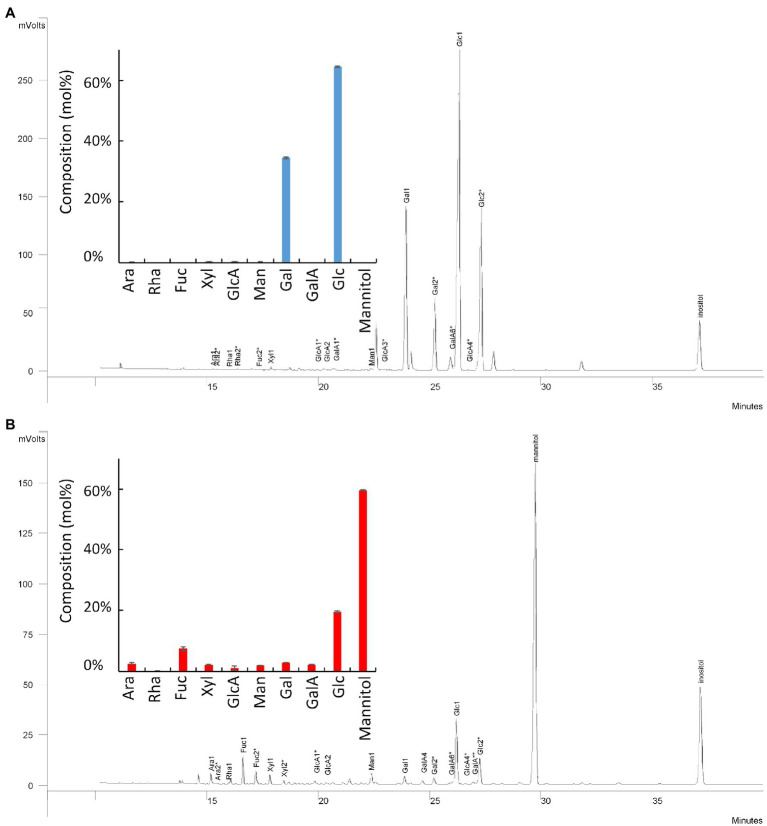
Monosaccharide composition of the two carbohydrate-based active ingredients: Active ingredients 1 (**A**, blue bars) and 2 (**B**, red bars). Ara, arabinose; Rha, rhamnose; Fuc, fucose; Xyl, xylose; GlcA, glucuronic acid; Man, mannose; Gal, galactose; GalA, galacturonic acid; Glc, glucose. Data are mean of three replicates.

A screen of an in-house bioactive carbohydrate-based natural extracts was conducted, and two products were selected regarding their abilities to enhance tomato pollen germination. P1 and P2 correspond to the water-solubilized bioactivators containing the carbohydrate-based active ingredients 1 and 2, respectively. Monosaccharide composition of P1 and the active ingredient 1 showed that the trifluoroacetic acid (TFA)-soluble fractions were composed of 72% Glc, 26% Gal, and 1.1% of GlcA (Figure 1A). The percentage of the other monosaccharides was lower than 0.2%. Monosaccharide composition of P2 and the active ingredient 2 revealed that they contained 64% of mannitol, 19.5% Glc, 6.9% Fuc, 2.6% Gal, and 2.3% Xyl (Figure 1B).

Bioactive capacities of the products were tested on the germination of tomato pollen grain at 22°C, and the optimal concentration for further investigation was determined at 2μg.ml^−1^. When pollen grain germinated at 22°C in the presence of 2μg.ml^−1^ of P1, the percentage of normal pollen tubes was 4.8% higher than in the control (Supplementary Table S1). P1 and P2 were then used at 2μg.ml^−1^. The individual effect of the active ingredients, the products, and a mix of P1 and P2 were then assessed on the percentage of germination upon cold or heat temperature (Supplementary Table S2). P1 and P2 showed a significant increase in the percentage of normal pollen tubes after 2h at 13°C, and P2 showed a significant increase in the percentage of normal pollen tubes after 2h at 8°C (Supplementary Table S2). However, a mix of the products or the use of the active ingredients was not more beneficial than the treatment with the water-solubilized active ingredient P1 and P2. Therefore, further investigations were all carried out with a final concentration of the active ingredient at 2μg.ml^−1^ in the products.

### Cold Temperatures Impact Germination of Tomato Pollen in Control Condition

**Figure 2:**
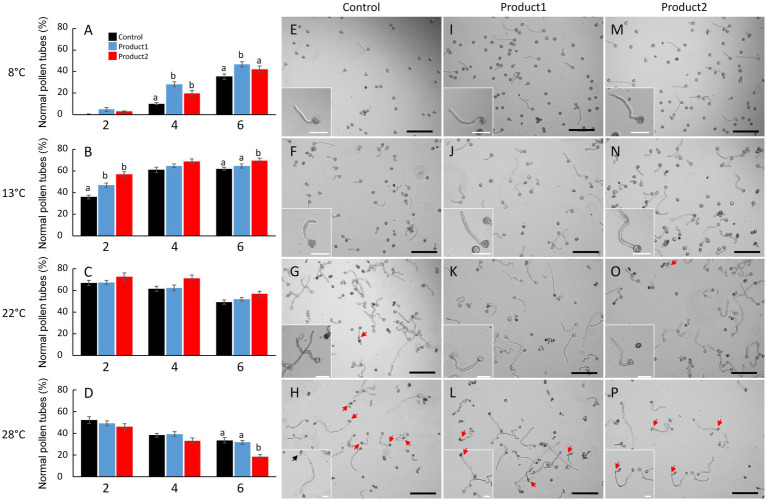
Impact of temperature and treatments on tomato pollen tube integrity and morphology. **(A–D)** Percentages of normal pollen tubes after 2, 4, and 6h of culture in the control condition (black bar) or in a medium supplemented with 2μg.ml^−1^ of product 1 (blue bar) or product 2 (red bar). **(E–P)** Representative pictures of pollen germination and pollen tube after 4h of culture in control condition **(E–H)** or in a medium supplemented with 2μg.ml^−1^ of product 1 **(I–L)** or product 2 **(M–P)**. Experiments were carried out at 8°C **(A,E,I,M)**, 13°C **(B,F,J,N)**, 22°C **(C,G,K,O)**, and 28°C **(D,H,L,P)**. Statistical analyses were carried out by one-way ANOVA and significant differences were analyzed by Dunnett’s multiple comparison test. Data are marked by different letters when significantly different to the control conditions at each temperature (*p*<0.05). Scale bars represent 200μm in the main pictures and 40μm in the inserts. Arrows indicate burst tubes.

Figure 2 shows the effect of temperature and treatments on pollen tube morphology and viability. At the optimal temperature (22°C) for *in vitro* growth of tomato pollen tubes, germination was fast with 67% of germinated pollen grains and viable pollen tubes after 2h of culture for the control (Figures 2C,G), and 15% of burst pollen tubes was observed (Supplementary Figure S1C). The number of normal and viable pollen tubes slowly decreased after 4 and 6h together with an increase in the rate of burst tubes (35% after 6h; Supplementary Figure S1C). Cold temperature (i.e., 13 and 8°C) had a strong effect on pollen germination rates. In fact, when pollen germination and pollen tube growth were performed at 13°C, 35% of pollen grains were germinated after 2h (Figure 2B) and 60% after 6h (Figure 2B). At 8°C, 10% of pollen grains were germinated after 4h, whereas 65% were germinated and produced a normal tube at 22°C (Figures 2A,C). Cold treatments at 8 and 13°C did not affect the morphology (Figures 2E,F) or the integrity of the pollen tube as the percentage of burst pollen tubes was below 10% after 6h of *in vitro* growth (Supplementary Figures S2A,B). At 28°C, pollen germination in the control conditions was strongly affected. The percentage of normal pollen tubes was 52% after 2h and~50% of pollen tubes were burst after 4h of culture (Figure 2H; Supplementary Figure 1D). After 6h, the rate of normal pollen tubes was less than 35% (Figure 2D) and numerous pollen tubes burst even after treatments with P1 and P2 (Figures 2D,H,L,P). Based on these data, the mild heat stress condition (28°C) was not further investigated and the study focused on the two cold temperatures (8 and 13°C) and the optimal temperature (22°C).

### P1 and P2 Promote Pollen Germination Under Cold Temperatures

Treatments with P1 or P2 promoted pollen germination at 8°C with a 3- and 2-fold significant increase in the levels of normal pollen tubes after 4h, respectively (Figure 2A). After 6h, higher levels of normal pollen tubes were observed in treated conditions, especially with P1 (Figure 2A). Indeed, 48% of pollen grains were germinated and pollen tubes were morphologically normal following P1 application when compared to the control (36%; Figure 2A). Pollen tube morphology was not affected by the products (Figures 2I,M). When pollen germination and pollen tube growth were performed at 13°C, the pollen tube morphology and integrity were also very well preserved (Figures 2J,N), and the rate of burst pollen tubes never exceeded 12, 11, and 9% after 6h for the control, P1 and P2, respectively (Supplementary Figure 1B). Treatments with P1 and P2 had positive effects on the number of normal pollen tubes, at 2, 4, and 6h of culture (Figure 2B). Particularly, after 2h of culture, P1 and P2 were significantly boosting the rate of normal pollen tubes (46 and 57%, respectively). In contrast, in the untreated control condition, the rate of normal pollen tubes was only 35% (Figure 2B). At 22°C, no significant effects were observed in treated pollen tubes (Figure 2C) when compared to the control conditions (60 and 48%, respectively, after 4 and 6h; Figure 2C). Pollen tubes treated with P1 and P2 behaved like the control with no apparent effect on pollen tube morphology (Figure 2K), and similar levels of viable pollen tubes after 2, 4, and 6h were observed (Figure 2C).

### P1 and P2 Stimulate Pollen Tube Growth at 8 and 13°C

**Figure 3:**
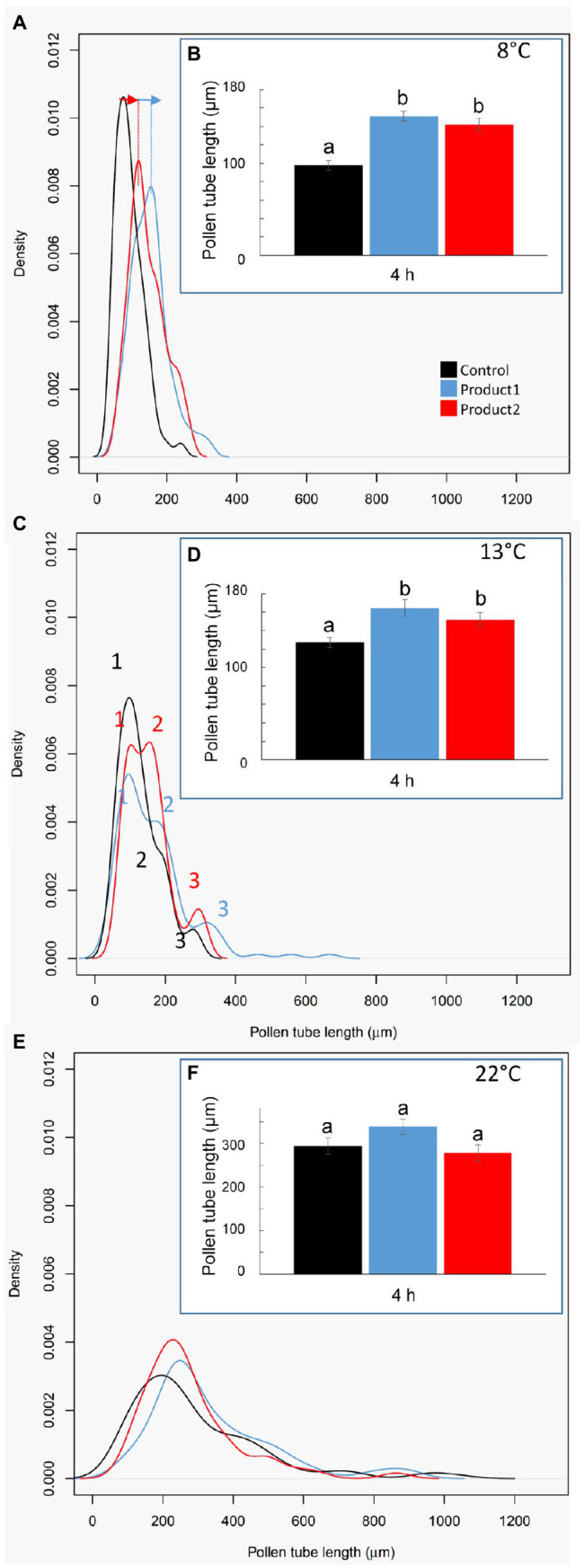
Impact of temperatures and treatments on tomato pollen tube length. **(A,C,E)** Distribution plot of pollen tube length after 4h of culture in the control condition (black line) or with 2μg.ml^−1^ of P1 (blue line) or P2 (red line) at 8 **(A)**, 13 **(C)**, and 22°C **(E)**. **(B,D,F)** Pollen tube length after 4h of culture in the control condition (black) or in a medium supplemented with 2μg.ml^−1^ of P1 (blue) or P2 (red) at 8 **(B)**, 13 **(D)**, and 22°C **(F)**. Statistical analyses were carried out by one-way ANOVA and significant differences were analyzed by Dunnett’s multiple comparison test. Data are marked by different letters when significantly different to the control conditions at each temperature (*p*<0.05).

The effect of the treatment with the two products was clearly visible when comparing the mean of pollen tube length and the density plot of the length of pollen tubes after 4h (Figure 3). Pollen tube length in control conditions increased with the temperature ranging from 100μm at 8°C, 120μm at 13°C to 280μm at 22°C (Figures 3B,D,F). At 8°C, the mean length of pollen tubes reached around 150μm when treated with P1 or P2 (Figure 3B). This significant increase in the length of pollen tubes was associated with a shift in the distribution of the pollen tube length population toward longer pollen tubes at 8°C as pointed out with blue and red arrows (Figure 3A). Similar results were obtained at 13°C. The mean length of the pollen tube in the control condition was around 125μm and was significantly higher (~150μm when treated with P1 or P2; Figure 3D). This increase in the pollen tube length in the treated conditions was probably due to the differences observed in the three main populations of pollen tubes referred as 1, 2, and 3 in Figure 3C. In the control condition, three populations of pollen tubes were clearly visible. The main population was at 100μm, an intermediate one at ~200μm, and the smallest one around 250μm (Figure 3C). With P1, the third population of pollen tubes shifts from 250μm to 300μm and the second one contained a higher density of pollen tubes than in the control (Figure 3C). When pollen tubes were treated with P2, there was no visible shift of the population toward longer pollen tubes but an increase in the population at 200μm and 250μm was observed (Figure 3C). At 22°C, the products P1 and P2 did not show any significant effects. The mean of pollen tube length was ~300μm in all the tested conditions (Figure 3F). A slight shift of the main population around 200μm in the control toward 250μm was observed in treated pollen with P1 and 2 but without significant statistical effect (Figures 3E,F).

Based on this, we decided to investigate molecular markers that may explain the stimulatory effects of the products at these two temperatures compared to the optimal at 22°C. First of all, in order to compare the control and the treated pollen tubes at the same developmental stage, we determined the D values (i.e., the duration required to reach 50% of the highest germination rate of the control condition for a given temperature). The D values are presented in Table 2, and the kinetics of the germination are presented in Supplementary Figure S2. D_8°_C, D_13°_C, and D_22°_C were 4h 40, 1h 45, and 0h 53 in the control condition, respectively (Table 2). Treatment with P1 or P2 decreased the D values at the tested temperatures with major effect at 8 and 13°C. D_8°_C and D_13°_C of pollen treated with P1 were 3h 05 and 1h 20 and 3h 45 and 1h with P2, respectively (Table 2).

As callose plug deposition (Qin et al., 2012) and callose inner cell wall (Parre and Geitmann, 2005), PMEs (Jiang et al., 2005; Tian et al., 2006) and NADPH oxidase were shown to be important during the fast pollen tube growth (Muhlemann et al., 2018), we further investigated these molecular markers. The callose plug number was determined, and the expression of several *CalS* and *Rboh* genes were studied by qRT-PCR. Total PME activity and PME isoform activities were determined by gel diffusion and by IEF followed by zymogram.

### The Number of Callose Plugs Is Slightly Higher Upon Treatment With P2

**Figure 4:**
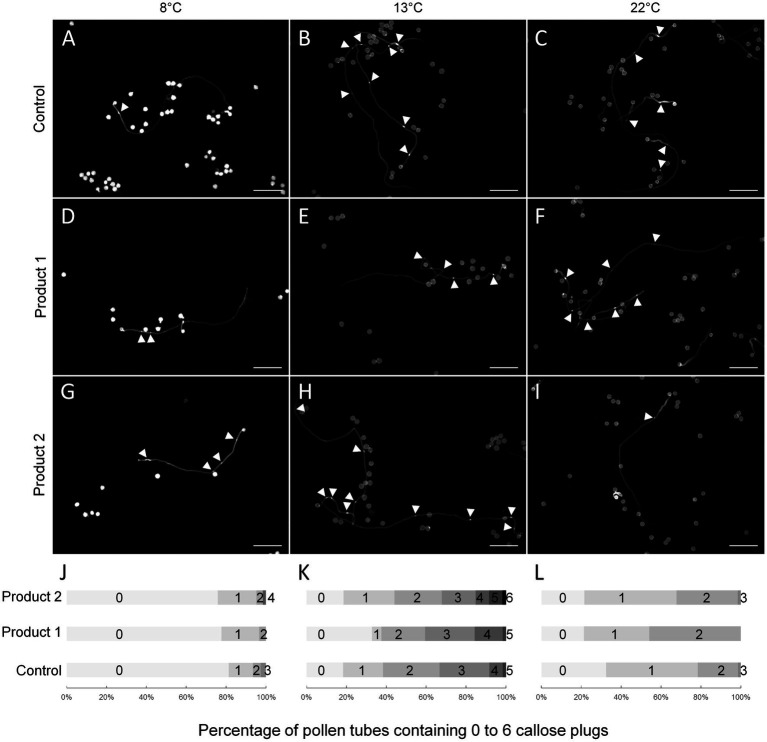
Impact of temperatures and treatments on callose plug deposition in tomato pollen tubes. **(A–I)** Representative pictures of aniline blue stained pollen tubes cultivated for 4*D at 8 **(A,D,G)**, 13 **(B,E,H)**, or 22°C **(C,F,I)** in the control condition **(A–C)** or in a medium supplemented with 2μg.ml^−1^ of P1 **(D–F)** or P2 **(G–I)**. **(J–L)** Percentage of pollen tubes containing 0 to 6 callose plugs in the control condition or in a medium supplemented with 2μg.ml^−1^ of P1 or P2 at 8 **(J)**, 13 **(K)**, and 22°C **(L)**. Arrowheads show callose plugs. Scale bars=200μm.

In fast-growing pollen tubes, callose plugs are regularly synthesized to maintain the vegetative cell in the apical region of the pollen tube. Pollen tubes were stained after a duration of culture corresponding to a 4-fold increase in the D values (4*D; Table 1), which is the necessary time for pollen tubes to synthesize callose plugs as at D, and no callose plug was detectable in any conditions. Thus, after 3h, 3h20, and 3h30 of culture at 22°C with P1, P2, and control conditions, the number of callose plugs ranged between 0 and 3 (Figures 4C,F,I,L). For the control condition, pollen tubes contained between 0–3 or 0–5 callose plugs at 4*D_8°_C and 4*D_13°_C, respectively (Figures 4A,B,J,K). The number of callose plugs was quite similar in P1 treated samples (Figures 4D,E,J,K) at 4*D_8°_C and 4*D_13°_C. A slight increase in the number of callose plugs was observed at 4*D_8°_C and 4*D_13°_C when pollen tubes were cultured with P2 (Figures 4G,H,J,K). Pollen tubes displaying four callose plugs at 4*D_8°_C and up to six callose plugs at 4*D_13°_C were only observed when treated with P2. In order to link the positive effect of P1 and P2 on pollen tube length under cold temperatures, the expression of putative tomato *CalS* genes was analyzed by qRT-PCR.

### Gene Expression of *Cals* and *Cals*-Like Genes Upon Treatment

*AtCalS5* was shown to be strongly expressed in pollen grains and pollen tubes and involved in callose deposition in the inner cell wall and plugs (Dong et al., 2005b; Abercrombie et al., 2011; Zaveska Drabkova and Honys, 2017). The tomato *CalS* candidate genes were selected using the protein sequence from the *Arabidopsis thaliana CalS5* gene (At2g13680) in a TBLASTN analysis (Altschul et al., 1997). The search was carried out in the NCBI transcript references sequences (refseq_rna) restricted to *Solanum lycopersicum* to retrieve transcripts corresponding to potential *CalS* cDNA sequences from tomato.

**Figure 5:**
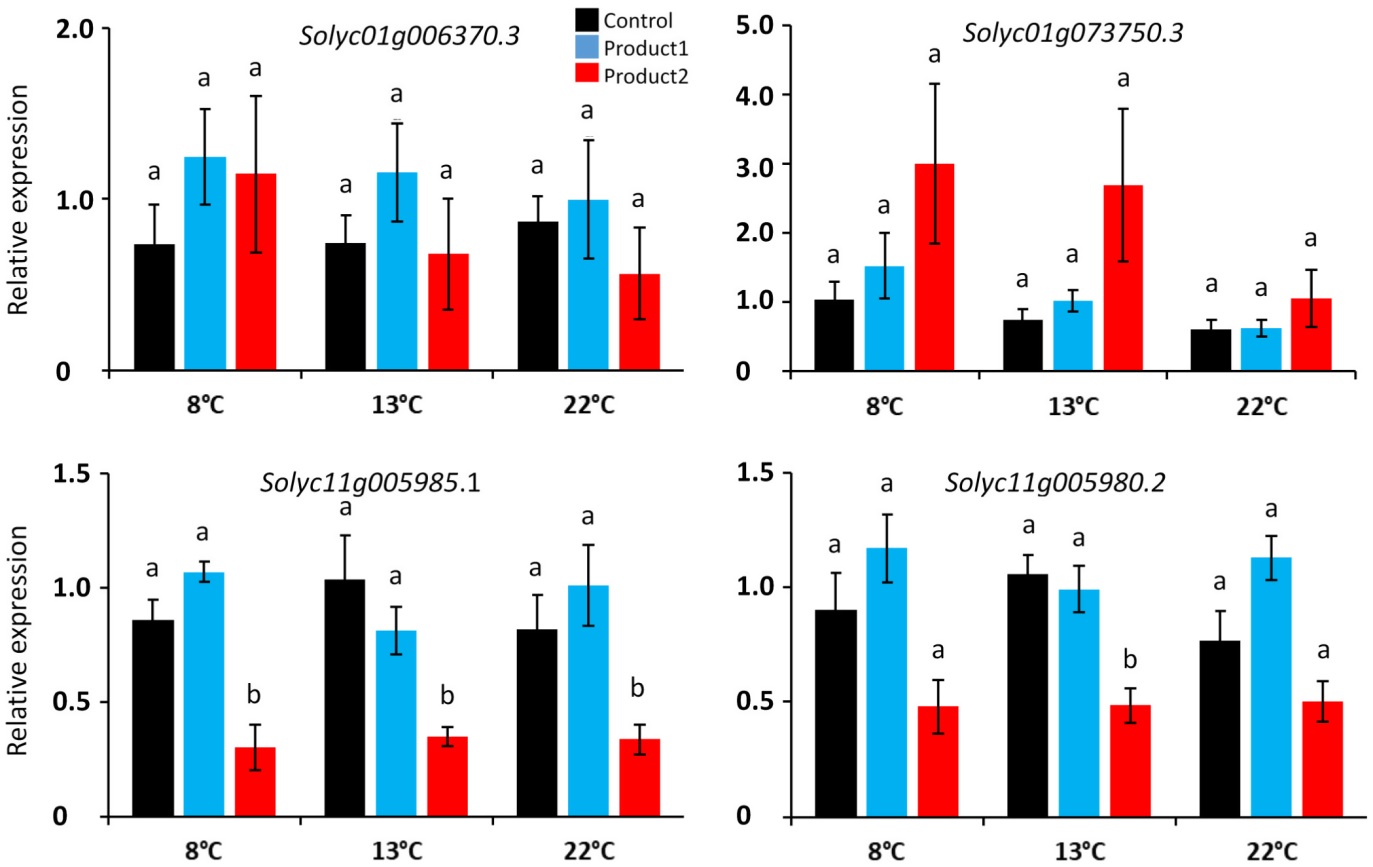
Relative expression of *CalS* and *CalS*-like genes in pollen tubes cultured for the duration corresponding to D_8°_C, D_13°_C, and D_22°_C. Different colors correspond to pollen tubes cultivated in control conditions (black), or in a medium supplemented with 2μg.ml^−1^ of P1 (blue) or P2 (red) at 8, 13, and 22°C. Relative expression corresponds to the mean of 6 biological replicates normalized against four reference genes (*EXP*, *LZ*, *EF1α*, and *CK2A*). Statistical analyses were carried out by one-way ANOVA and significant differences were analyzed by Dunnett’s multiple comparison test. Data are marked by different letters when significantly different to the control conditions at each temperature (*p*<0.05).

Finally, after validation of primer pairs, four *CalS* gene sequences were used for the qRT-PCR analyses: *Solyc01g006370.3*, *Solyc01g73750.3*, *Solyc01g006370.3*, *Solyc11g005980.2*, and *Solyc11g005985.1*. The effect of cold was not accompanied by strong variations in the expression of the *CalS* genes in the control condition (Figure 5). Treatments with P1 or P2 did not contribute to a measurable modification of the expression of the *CalS* genes except for the treatment with P2 on *Solyc11g005985.1* at 8, 13, and 22°C and *Solyc11g005980.2* at 13°C (Figure 5). These results can be explained by the heterogeneity of the gene expression among the six biological replicates (Supplementary Figure S3) except for *Solyc11g005980.2* and *Solyc11g005985.1*, for which the expression was very homogeneous (Supplementary Figure S3).

### Cold Temperatures Modify PME Isoform Activity

**Figure 6:**
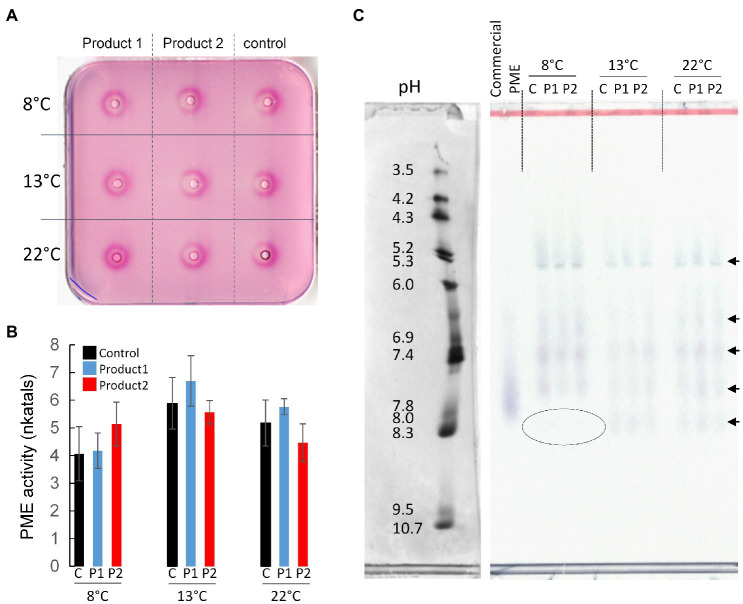
Determination of total PME activity and separation of the different isoforms. **(A,B)** Gel diffusion assay, and quantification of total PME activity expressed as nkatals in 20μg of proteins extracted from pollen tubes cultivated for 4*D at 8, 13, and 22°C in the control condition (black) or in a medium containing 2μg.ml^−1^ of P1 (blue) or P2 (red). **(C)** Zymogram after IEF showing the different PME isoform activities. A citrus commercial PME was used as positive control. A volume of protein corresponding to 0.05units of PME activity (U) was loaded in each well. C, control. Arrows indicate the main PME isoforms. The left in **(C)** corresponds to the pI markers of the native gels stained with coomassie blue.

As PMEs are key enzymes implicated in the remodeling of pectin during pollen tube growth, the total PME activity was assessed by gel diffusion and the profile of the different active isoforms of PMEs were detected with zymogram after IsoElectric Focalization (IEF). Interestingly, in the control condition, pollen cultured under cold temperature, total PME activities did not significantly change and were 4, 6, and 5.2 nkatals in 20μg of proteins at 8, 13, and 22°C, respectively (Figures 6A,B). The treatments with P1 or P2 did not significantly modify the total PME activities compared to the control. They were 4, 6.5, and 6 nkatals in 20μg of proteins with P1, whereas they were 5, 5.5, and 4.5 nkatals with P2 at 8, 13, and 22°C, respectively (Figures 6A,B). PME isoforms were separated by IEF based on their isoelectric points and their activities were visualized on a pectic gel. In optimal condition (22°C), the PME activities were mostly resulting from five isoforms (Figure 6C). Two acidic isoforms, at pH 5.3 and between 6.0 and 6.9, a neutral (pH 7.4), and two alkaline isoforms (between pH 7.4 and 7.8, and 8.3) were detected. There was no clear differences between the treatments at 22°C and the results obtained at 13°C with or without the addition of P1 or P2 (Figure 6C). Interestingly, at 8°C, the most alkaline PME isoform (pH 8.3) was not detected in the control and the treated conditions (Figure 6C). The lack of this PME isoform or its inhibition under cold temperatures seemed to be compensated by higher activities of the other isoforms, particularly the isoforms at pH 5.3, 7.4 and the one between 7.4 and 7.8 (Figure 6C).

### Cold Temperatures Modify NADPH Oxidase Expression in Treated Samples

**Figure 7:**
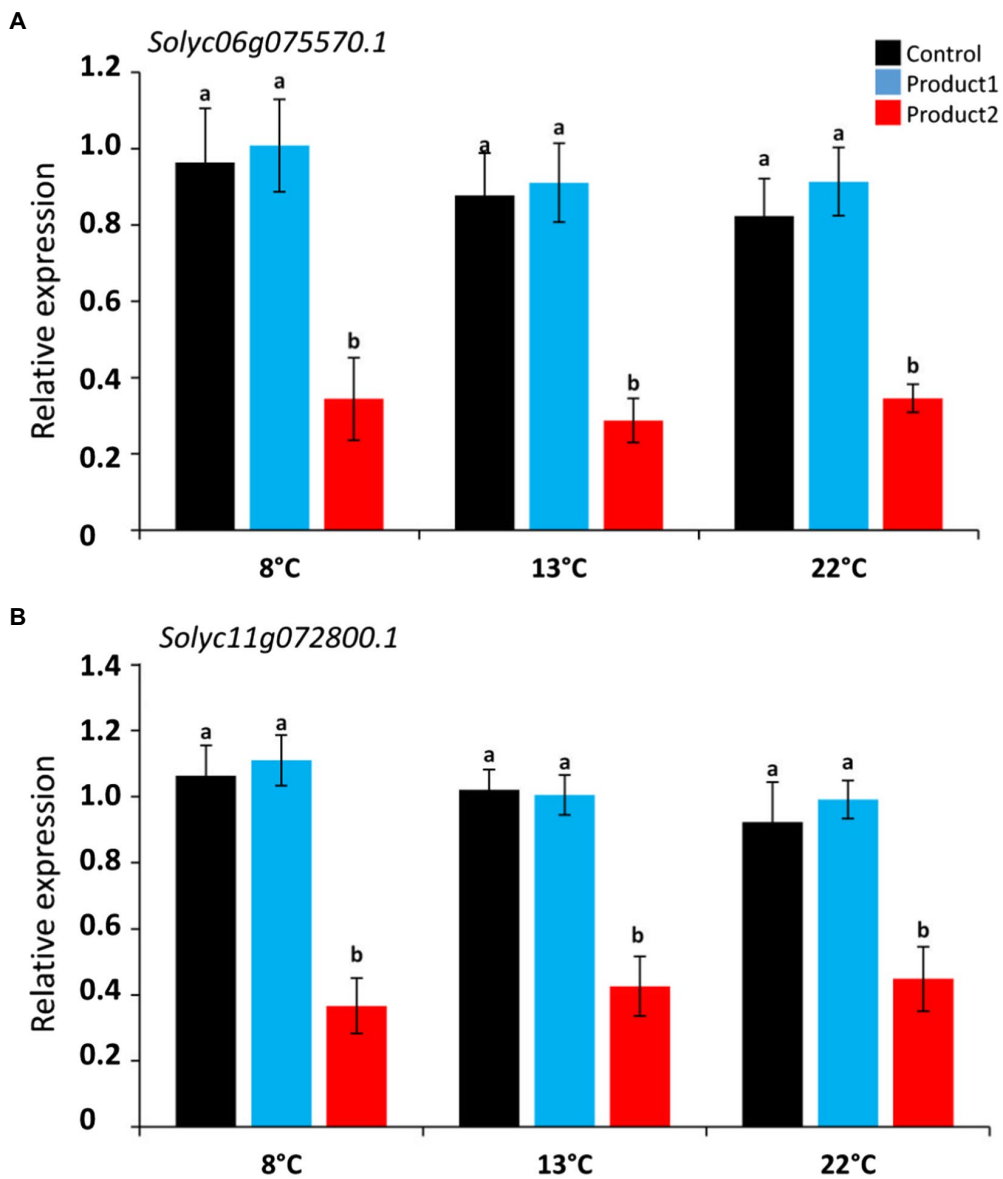
Relative expression of NADPH oxidase genes: *Rboh1* and *Rboh2* cultivated for the durations corresponding to D_8°_C, D_13°_C, and D_22°_C. **(A)** Relative expression of *Rboh1* (*Solyc06g075570.1*). **(B)** Relative expression of *Rboh2* (*Solyc11g072800.1*). Different colors correspond to pollen tubes cultivated in control condition (black), or in a medium supplemented with 2μg.ml^−1^ of P1 (blue) or P2 (red) at 8, 13, and 22°C. Relative expression corresponds to the mean of six biological replicates normalized against four reference genes (*EXP*, *LZ*, *EF1α*, and *CK2A*). Statistical analyses were carried out by one-way ANOVA, and significant differences were analyzed by Dunnett’s multiple comparison test. Data are marked by different letters when significantly different to the control conditions at each temperature (*p*<0.05).

Pollen tube growth is also coordinated by intracellular signals. For example, O_2_^•−^ and H_2_O_2_ were shown to be key elements for pollen tube growth (Potocký et al., 2007; Boisson-Dernier et al., 2013). Using *Arabidopsis thaliana* pollen-expressed *Rboh* (*RbohH* At5g60010 and *RbohJ* At3g45810; Boisson-Dernier et al., 2013), we retrieved two *Rboh* in the tomato genome *Solyc06g075570.1* (*Rboh1*) and *Solyc11g072800.1* (*Rboh2*) using TBLASTN analysis. Their relative expressions in cold conditions and the impact of P1 or P2 were followed. Results are shown in Figure 7 and Supplementary Figure S4. Cold temperatures did not significantly modify the expression of *Rboh1* and *Rboh2* in the control condition (Figure 7; Supplementary Figure S3). Treatment of pollen tubes with P1 did not have any effect on *Rboh* gene expression when compared to the control (Figure 7; Supplementary Figure S3). In contrast, treatment of pollen with P2 strongly and significantly reduced the expression of *Rboh1* at all the tested temperatures (Figure 7A; Supplementary Figure S3) and *Rboh2* (Figures 7B; Supplementary Figure S3).

## Discussion

The deleterious effects of cold stresses were previously studied on a Peruvian ecotype of tomato (Zamir et al., 1981) and authors showed that pollen grain germination was inhibited below 5°C (Zamir et al., 1981). In our study, using *Solanum lycopersicum* var. *cerasiforme* cv. WVa106 as an experimental model, we confirmed that tomato pollen grains were very sensitive to cold temperature and no germination was observed after 16h of culture at 4°C. Moreover, we showed that moderate cold stresses have a strong effect during the germination of pollen grains. As a matter of fact, the germination rate decreased and only one-third of the pollen grains were germinated after 6h at 8°C. Moreover, pollen tubes were also shorter when compared to the control condition, at 22°C. These results are in agreement with previous studies showing the effect of cold temperatures on pollen germination and pollen tube growth. For example, cold temperatures were shown to impact pollen germination and pollen tube length in *Trifolium repens* cultivated at 10 or 14°C instead of 22°C (Jakobsen and Martens, 1994) and chickpea cultivated in cold night regime (5°C at night, 15°C during the day; Srinivasan et al., 1999). The reduction in the pollen tube length prevents the pollen tubes to reach the ovules and leads to a reduction in seed production (Jakobsen and Martens, 1994; Zinn et al., 2010).

Numerous molecules secreted by the female tissues are known to act on pollen germination, pollen tube growth, and guidance during sexual reproduction. Pollen tubes can be guided by unusual amino acids like D-serine (Michard et al., 2011) or polypeptides such as chemocyanin (Kim et al., 2003), plantacyanin (Dong et al., 2005a), defensin-like (LURE; Okuda et al., 2009), *Zea mays* EGG APPARATUS 1 (ZmEA1; Márton et al., 2005), and others reviewed by Kanaoka and Higashiyama (2015) and Johnson et al. (2019). Glycomolecules were also implicated in promoting pollen tube growth and guidance such as arabinogalactan proteins (Wu et al., 1995; Mollet et al., 2002; Coimbra and Duarte, 2003; Nguema-Ona et al., 2012; Pereira et al., 2016; Leszczuk et al., 2019) and arabinogalactan glycomodules (Hou et al., 2016; Mizukami et al., 2016; Jiao et al., 2017). In contrast, they could be repelled *in vitro* by nitric oxide (Prado et al., 2004). Flavonols were also shown to improve pollen germination (Mo et al., 1992; Ylstra et al., 1992). All these molecules are naturally synthesized by the pistil, and only a few studies have shown the effect of exogenous application of natural extracts on pollen germination with the exception of pyrroloquinoline quinone on *Lilium* pollen germination (Xiong et al., 1988) and the exogenous supply of polyamines such as spermidine on many species (Sorkheh et al., 2011; Rodriguez-Enriquez et al., 2013).

In this study, we used two carbohydrate-based natural extracts to stimulate pollen germination and pollen tube growth *in vitro* under cold temperature. A first one was mainly composed of galactose and glucose (P1), and the second was mainly made of mannitol together with glucose and fucose (P2). The two carbohydrate-based natural products significantly increased pollen germination rates by promoting the number of normal and viable pollen tubes. They also promoted pollen tube growth. The length of pollen tubes is generally correlated with the number of callose plugs and in *A. thaliana*, pollen tubes without a callose plug are shorter than those with a callose plug (Qin et al., 2012). Moreover, in *Hibiscus moscheutos*, the number of callose plugs was used as an indicator of pollen tube growth rate (Snow and Spira, 1991a,b), and the absence of callose plug was correlated with the inability of pollen tubes to reach the ovule in Petunia (Guyon et al., 2004). Finally, it has been proposed that angiosperm pollen tubes that synthesize callose plugs have an evolutionary advantage over gymnosperms, which do not, leading to faster-growing pollen tubes, thus reducing considerably the timing intervals between pollination and fertilization (Williams, 2008; Abercrombie et al., 2011; Dehors et al., 2019). Based on that it was legitimate to search for a correlation of the positive effect of P1 or P2 on callose deposition. In our study, there was no significant statistical differences regarding the number of callose plugs between the control and the biostimulated pollen tubes, whereas a small population of pollen tubes had more callose plugs in the treated condition compared to the control. As suggested by Qin et al. (2012), we searched for differences in the expression of *CALS* genes that regulate callose wall synthesis and plug deposition in order to link their expression to pollen tube length or growth rate. Surprisingly, the expression of two putative *CALS* genes (*Solyc11g005985.1* and *Solyc11g005980.2*) were reduced in pollen tubes treated with P2, whereas this treatment was clearly beneficial for the length of pollen tubes at 8 and 13°C. Treatment with P1 did not induce any differences in the expression of *CALS* although P1 also had a positive effect on the pollen tube length. It is then difficult to link the synthesis of callose with the pollen tube length in *Solanum lycopersicum* under cold temperature. However, the *cals5* pollen mutant of *A. thaliana* had no callose plug but displayed normal growth both *in vitro* and *in vivo* (Nishikawa et al., 2005) suggesting that callose is not the only polymer influencing the normal pollen tube growth.

In fact, pectins, more specifically HG, and their modifications are thought to control the mechanical properties of the cell wall during pollen tube growth (Chebli et al., 2012; Mollet et al., 2013; Leroux et al., 2015; Dehors et al., 2019). It is generally established that HG are deposited in the cell wall under a highly methylesterified form (Zhang and Staehelin, 1992; Li et al., 1997; Sterling et al., 2006) and are then de-esterified through the activity of PMEs within the cell wall (Wolf et al., 2009) in the sub-apical zone. This tight regulation is thought to control the stiffness of the cell wall between the tip (elasticity) and the shank (rigidity) to maintain the cylindrical shape of fast-growing pollen tubes (Taylor and Hepler, 1997; Wolf and Greiner, 2012; Dehors et al., 2019). PME activity is crucial during pollen germination (Leroux et al., 2015) and also during pollen tube growth (Jiang et al., 2005; Tian et al., 2006). The functional disruption of *PME48* (At5g07410) resulted in a strong delay of *in vitro* and *in vivo* germination, whereas functional disruption of *VGD1* (*PME5*, At2g47040) resulted in burst pollen tubes *in vitro* and a strong reduction in male fertility and seed set. Another PME, (PPME1, At1g69940) was shown to be involved in the determination of the shape of the pollen tube and the growth rate (Tian et al., 2006). We, therefore, tried to link the treatment and the length of the treated pollen tubes with PME activity but no clear correlation was made. However, our data show that the cold condition at 8°C modified the PME profiles with the absence of a basic isoform activity. This unexpected effect could be the result of differential expression of *PME* genes in response to cold. In *A. thaliana* pollen grains, cold treatments modified gene expressions and a 10-fold increase of two *PME* genes (*PME48* and *PME49*) was observed in cold treated (72h at 0°C) pollen grains (Lee and Lee, 2003). We cannot exclude that in tomato, PMEs are differentially expressed under cold temperatures. Cold treatment might also have modified the interaction of PMEs with their inhibitors (PMEI; Hocq et al., 2017). The consequences of PMEI-PME interactions are diverse. Exogenous application of PMEI either drastically alters (Woriedh et al., 2013; Paynel et al., 2014; Hocq et al., 2017) or promote pollen tube growth depending on the pH range needed for inhibition (Hocq et al., 2017). The overexpression of *AtPMEI-1* or *AtPMEI-2* induced a 20% increase of root length (Lionetti et al., 2007). Even if the role of PMEI during cold stresses have not been extensively studied, it was shown in pepper (*Capsicum annuum*) that overexpression of *CaPMEI1* in *A. thaliana* could enhance resistance to oxidative and drought stresses (An et al., 2008). Moreover, *A. thaliana* plants overexpressing *AtPMEI13* or the blue mustard (*Chorispora bungeana)* expressing *CbPMEI1* showed decreased freezing tolerance, increased salt tolerance, and displayed longer roots than wild-type plants (Chen et al., 2018) revealing pleiotropic effects of PMEI. A recent study on tomato pollen subjected to heat stress has revealed that pollen mostly responds to heat by modulating the proteome rather than the transcriptome (Keller et al., 2018). We can then hypothesize that PMEI and their interactions with PMEs can be modified during cold treatments.

Pollen tube growth rate is obviously dependent on the cell wall synthesis, deposition and remodeling but many other signals are also involved during growth. Tip-localized ROS are essential for pollen tube growth (Potocký et al., 2007; Speranza et al., 2012; Boisson-Dernier et al., 2013). NADPH oxidases produce O_2_^•−^, which are converted to H_2_O_2_ by superoxide dismutases. The inhibition of NADPH oxidases using diphenilene iodonium chloride (DPI) on tobacco pollen tubes resulted in growth arrest, which was rescued by addition of exogenous H_2_O_2_ (Potocký et al., 2007). In *Arabidopsis*, two NAPDH oxidases (*RBOHh* and *RBOHj*) are expressed in the pollen tube. Almost all pollen tubes of the double mutant *rbohh/rbohj* burst rapidly *in vitro* (Boisson-Dernier et al., 2013; Kaya et al., 2014). Exogenous supply of Ca^2+^ increased tip-localized ROS and *in vitro* NADPH oxidase activity is stimulated by Ca^2+^ (Potocký et al., 2012) suggesting that NADPH oxidase activity is modulated *in vivo* by Ca^2+^. Low temperature stress was also shown to enhance the transcripts, proteins, and activities of different ROS-scavenging enzymes (Prasad et al., 1994; Saruyama and Tanida, 1995; O’Kane et al., 1996) and the production of ROS by NADPH oxidases (Suzuki and Mittler, 2006). In our study, cold did not affect *Rboh* expression in tomato pollen tubes in the control condition or with P1. However, P2 strongly decreased the expression of the two *Rboh* genes. This reduction in *NADPH oxidase* gene expression was correlated with a higher number of viable pollen tubes compared to the control. Taken together, our results showed that cold temperature affected tomato pollen tube growth. The two products tested were capable to reduce the effect of cold. The beneficial effects of the products are not clearly linked with the modulation of the classical molecular markers implicated in pollen tube growth. However, P2 had a strong effect on *Rboh* expression and thus probably on ROS production and this effect could explain the higher number of viable pollen tubes. Recently, a study has shown that the brassinazole signaling regulator *BRASSINAZOLE RESISTANT 1* (*BZR1*) could directly bind to the promoter of *Rboh* in tomato (Yan et al., 2020). Thus, it could be interesting to further investigate the link between the treatment and brassinosteroids.

Finally, it has been described that cold stress could induce a reduction in bioactive gibberellins in cold-susceptible anthers (Sakata et al., 2014). In fact, the regulation of gibberellin homeostasis perception by SlGRAS40 was shown to be involved in plant reproduction during abiotic stresses (Liu et al., 2017). Moreover, Huang et al. (2017) showed that the miR171-GRAS module (involved in gibberellin perception and signaling) and its modulation impaired fertilization in tomato. Additionally, in tomato, overexpression of *KNOX*, which is involved in gibberellin perception, led to a strong increase in pollen tube length together with the increase in cell wall modifying genes such as pectate lyase (Yan et al., 2019). Moreover, cold stress can also induce an accumulation of soluble sugars including oligosaccharides (fructans, raffinose oligosaccharide family) in cold-tolerant anthers (Sharma and Nayyar, 2016) leading to viable pollen. Sugars can play multiple functions in protecting plants such as ROS scavenging, membrane stabilization, and signaling leading to the induction of cold tolerance (Sharma and Nayyar, 2016). Thus, other possible explanations for the positive effect of those two carbohydrate-based products during cold treatment are that they (i) may have a direct effect on scavenging OH^•^ resulting in the formation of new oxidized oligosaccharides/sugars (Tarkowski and Van den Ende, 2015; Tenhaken, 2015), which may be internalized and used as a primary energy source, (ii) may directly stabilize the membrane fluidity (Tarkowski and Van den Ende, 2015) at the pollen tube tip allowing a faster growth, (iii) may serve, depending on the chemical structure, as signaling molecules for pollen germination (Hirsche et al., 2017) and/or pollen tube growth, or (iv) may help to regulate ROS homeostasis by scavenging ROS, therefore, allowing a greater expression and activity of NADPH oxidase promoting a fast growth without the deleterious effect of ROS, like flavonol does in tomato pollen tubes (Muhlemann et al., 2018).

Interestingly, the products did not affect the general functioning of the reproductive cell (callose plug, pectin remodeling enzymes) but seems to positively modify the ROS perception and management. Even if the molecular targets of these compounds are not known yet and that further works are needed to precise their mode of action, the current study confirms that the use of natural carbohydrate-based compounds can effectively promote germination and growth of tomato pollen tube under cold conditions. This is of the utmost importance as it is known that short cold periods during flowering can alter dramatically the fertilization process and subsequently the production of seeds and fruits (Zinn et al., 2010; Gammans et al., 2017). Even if climate changes are now accepted, the effects on crop production are much more complex to predict than a global increase in the temperature everywhere on Earth (Lobell and Gourdji, 2012). Major crops could be geographically displaced as predicted for the date palm trees (Shabani et al., 2012). In fact, climate changes in the agricultural regions will result in more extreme temperature fluctuations (Solomon et al., 2007) and could threat pollinators (Giannini et al., 2017). In many actual cases, certain crops are now cultivated in other regions to avoid the effect of increasing temperatures (Zinn et al., 2010). This is the case for soybean (Dong et al., 2004) and rice (Oliver et al., 2005) and others. This results in farming at higher altitude with possible colder temperatures during plant reproduction (Dong et al., 2004; Oliver et al., 2005). Therefore, the possibility to develop new biostimulants that can improve pollen germination and pollen tube growth in deleterious conditions could represent a substantial progress for modern agriculture in order to face climate changes.

In conclusion, this study reveals that the two carbohydrate-based compounds assisted *in vitro* pollen germination and tube growth under cold temperatures and may possibly have a beneficial effect on *in vivo* germination. However, further field trials under real conditions are necessary to confirm the data obtained *in vitro*.

## Data Availability Statement

All datasets presented in this study are included in the article/Supplementary Material.

## Author Contributions

FC, J-CY, EN-O, J-CM, FJ, and AL designed the research. AL, J-CM, FJ, and EN-O analyzed the data and wrote the paper. FC, J-CY, EN-O, and FJ provided the products. FL, GP, and ST performed pollen culture, callose staining, and image analyses, and collected the samples for molecular and biochemical analyses. M-CK-M and NA designed the gene expression experiments. FL, GP, ST, and NA performed and analyzed the gene expression experiments. BG designed, performed, and analyzed the gas chromatography experiment and data. AM designed and analyzed the biochemical experiment with the help of FL, GP, and ST. All authors contributed to the article and approved the submitted version.

## Funding

This work was funded by CMI Roullier and by Université de Rouen Normandie (UNIROUEN, Normandie Université).

## Conflict of Interest

The authors declare that the research was conducted in the absence of any commercial or financial relationships that could be construed as a potential conflict of interest.

## Publisher’s Note

All claims expressed in this article are solely those of the authors and do not necessarily represent those of their affiliated organizations, or those of the publisher, the editors and the reviewers. Any product that may be evaluated in this article, or claim that may be made by its manufacturer, is not guaranteed or endorsed by the publisher.

## Footnotes

^1^ https://www.ncbi.nlm.nih.gov/tools/primer-blast/
