# Supplementary material for: Two Carbohydrate-Based Natural Extracts Stimulate in vitro Pollen Germination and Pollen Tube Growth of Tomato Under Cold Temperatures
Source: Front Plant Sci. 2021 Oct 7;12:552515. doi: 10.3389/fpls.2021.552515 (PMC8529017; doi:10.3389/fpls.2021.552515)
Supplement: Supplementary file 1 [file Data_Sheet_1.PDF]

Supplementary Material

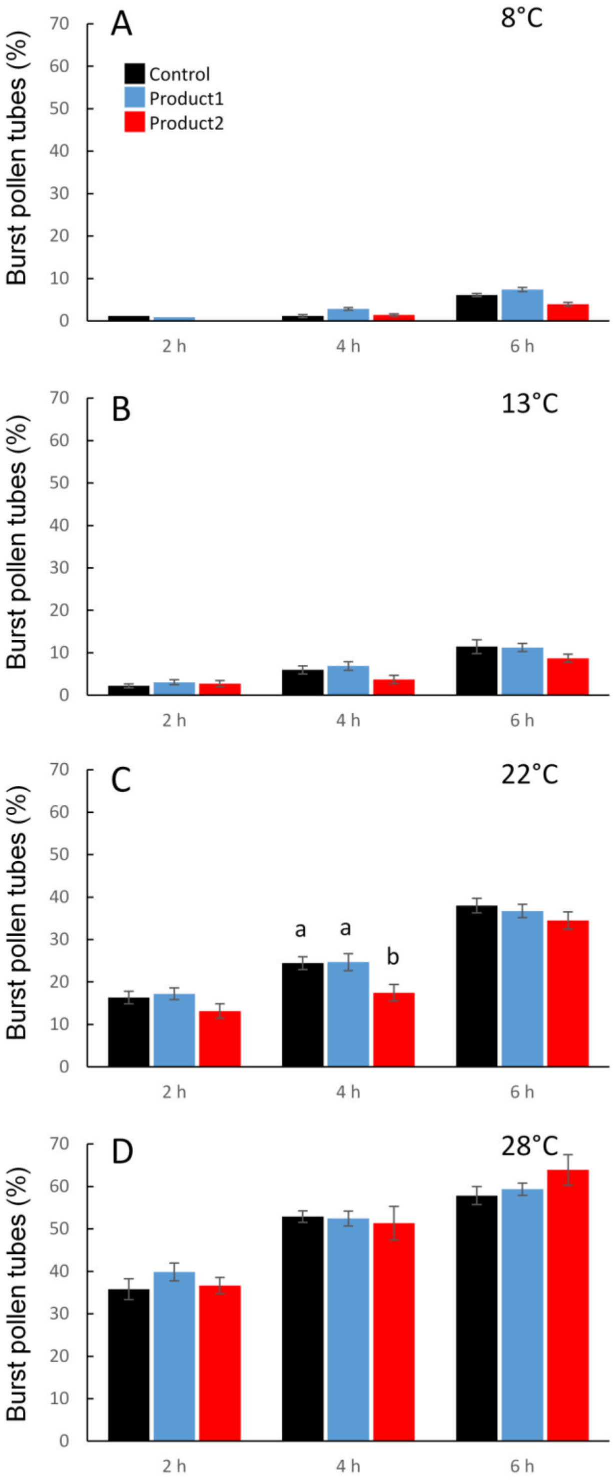

Supplementary Figure S1: Impacts of treatments and temperatures on tomato pollen tubes integrity. A-D, percentages of burst pollen tubes after 2, 4 and 6 h of culture in the control condition (black bar) or in a medium supplemented with 2 $\mu g.mL^{-1}$ of P1 (blue bar) or P2 (red bar). Experiments were carried out at 8°C (A), 13°C (B), 22°C (C) and 28°C (D). Statistical analyses were carried out by one way ANOVA and significant differences were analyzed by Dunnett's multiple comparison test. Data are marked by different letters when significantly different with respect to control conditions at each temperature ($p<0.05$).

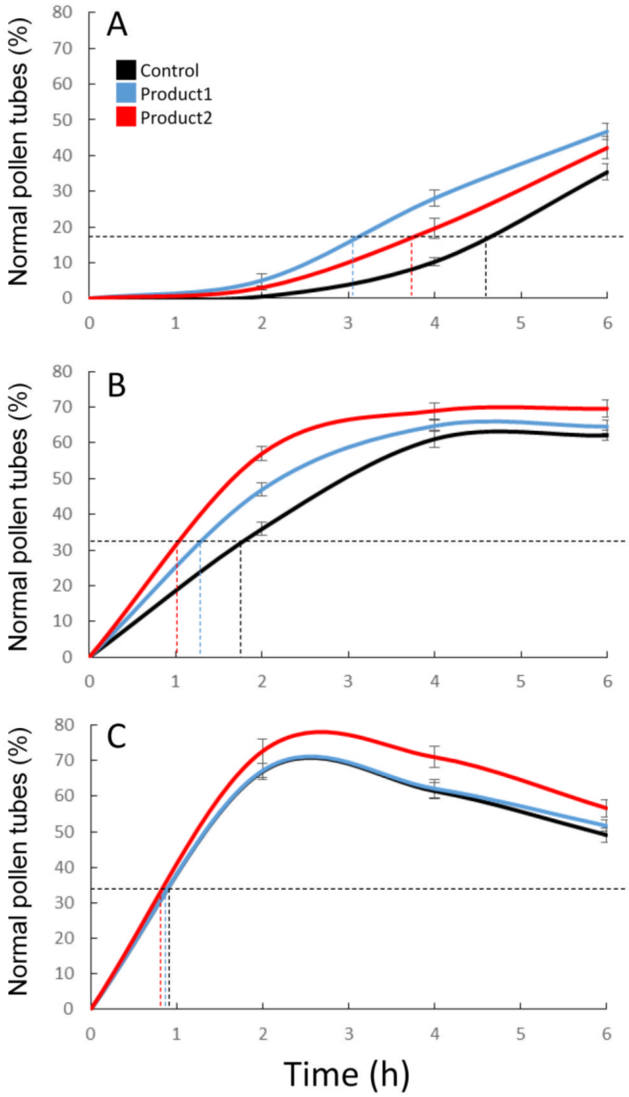

Supplementary Figure S2. Graphical representation of D (*i.e.* the duration necessary to reach 50% of the highest germination rate of the control for a given temperature). A, B, C, kinetic of germination of normal pollen tubes in the control condition (black line) or in a medium supplemented with 2 µg.mL^{-1} of P1 (blue line) or P2 (red line) at 8 (A), 13 (B) and 22°C (C). Horizontal dashed line represents 50 % of the maximum percentage of viable pollen tubes obtained in control condition and vertical projection indicates the D.

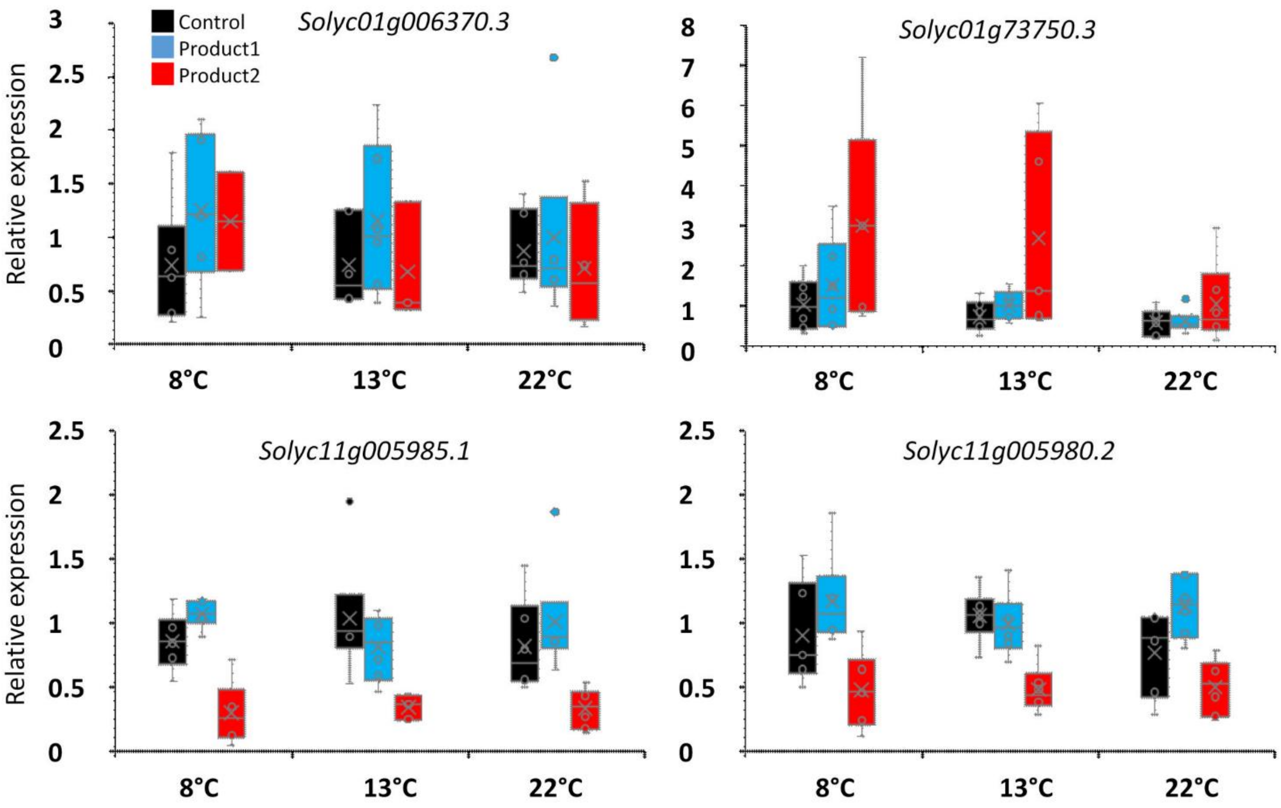

Supplementary Figure S3. Boxplots showing variation in the relative expression of *CalS* genes between the six biological replicates. Different colors correspond to pollen tubes cultivated in the control condition (black), or in a medium supplemented with 2 µg.mL^{-1} of P1 (blue) or P2 (red) at 8, 13 and 22°C. Relative expression for each replicate was normalized against four reference genes (*EXP*, *LZ*, *EF1α* and *CK2A*).

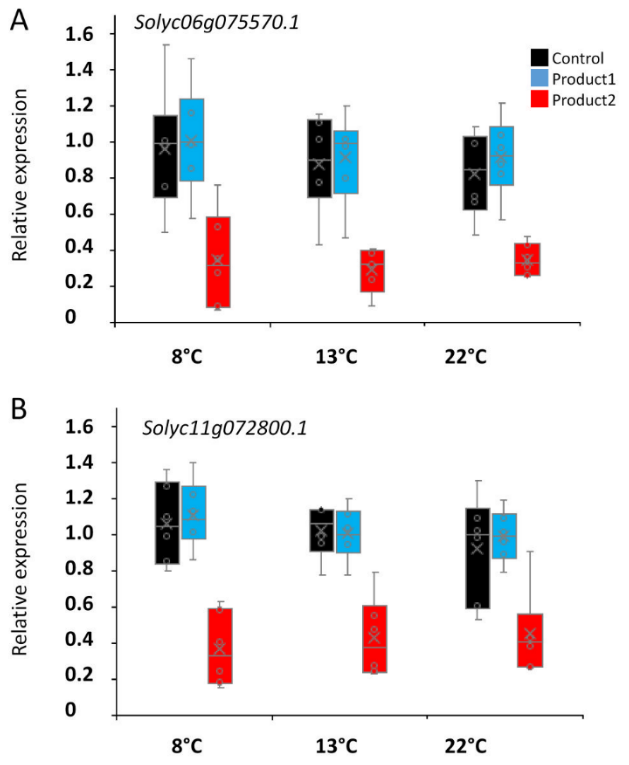

Supplementary Figure S4. Boxplots showing variation in the relative expression of *Rboh* genes between the six biological replicates. (A), Relative expression of *Rboh1* (*Solyc06g075570.1*). (B-C), relative expression of *Rboh2* (*Solyc11g072800.1*) with two different sets of primers. Different colors correspond to pollen tubes cultivated in the control condition (black), or in a medium supplemented with 2 µg.mL^{-1} of P1 (blue) or P2 (red) at 8, 13 and 22°C. Relative expression for each replicate was normalized against four reference genes (*EXP*, *LZ*, *EF1α* and *CK2A*).

Supplementary Table S1. dose response effects of Product 1 on the germination of tomato pollen grain at 22°C. Results are expressed as the gain or the loss of percentage compared to the control. * indicates significant difference between treatment and control as determined by Dunnett's test (P<0.05).

Concentration (µg ml^{-1})	Ungerminated	Normal pollen tubes	Burst pollen tubes
1	+2.93±1.12	-2.00±1.04	-0.83±0.26
2	-8.11±0.58*	+4.86±0.49*	+3.34±0.26*
5	-3.95±0.83*	+2.29±1.03	+1.77±0.49
10	-11.46±0.60*	+3.96±0.65*	+7.60±0.40*
75	+5.29±0.99*	-18.11±0.72*	+12.92±0.79*
150	+5.17±0.95*	-24.39±0.77*	+19.32±0.43*
300	+63.84±1.36*	-61.77+0.75*	-1.97±0.64

Table S2: effect of Product 1 and 2 a mix of Product 1 and Product2 (mix) and the active plant extract of Product 1 and Product 2 (extract 1, extract 2). Final concentration of the extract is 2 µg ml^{-1}. Experiments were conducted at 8, 13, 22 and 28°C for 2 h. * indicates statistically significant differences between control and treatment as determined by one way ANOVA followed by Dunnett's test (P <0.05).

Temperature	Treatment	Ungerminated pollen grains	Normal pollen tubes	Burst pollen tubes
8°C	Control	98.30±0.57	0.52± 0.28	1.18±0.36
	P1	94.08±1.69*	5.05± 1.71	0.86±0.30
	P2	96.67±0.61	3.02± 0.55	0.17±0.27
	Mix	99.45±0.30	0.55± 0.30	0.00±0.00
	Extract1	97.44±0.81	1.81± 0.782	0.74± 0.43
	Extract2	96.45±1.00*	2.89± 0.82	0.65± 0.37
13°C	Control	61.87±1.94	35.91± 1.82	2.22± 0.47
	P1	50.03±1.85*	46.91± 1.82*	3.05±0.59
	P2	40.26±2.14*	57.02±2.03*	2.71±0.73
	Mix	45.60± 1.88*	52.99± 1.72*	1.41±0.56
	Extract1	48.64± 2.98*	49.59± 2.65*	1.76±0.68
	Extract2	39.88±3.71*	58.74±3.33*	1.37±0.56
22°C	Control	16.71±2.00	66.95±2.24	16.34±1.48
	P1	15.55±2.07	67.23±1.94	17.22±1.40
	P2	14.15±3.33	72.69±3.36	13.15±1.69
	Mix	14.74±3.71	71.42±2.87	13.84±1.78
	Extract1	22.88±5.08	62.90±3.74	14.22±1.93
	Extract2	15.52±2.83	70.27±2.91	14.20±1.50
28°C	Control	11.87± 1.63	52.36±2.92	35.77±2.46
	P1	11.26±1.45	49.33±2.30	39.87±2.08

P2	17.19±1.95	46.17±2.89	36.64±1.91
Mix	16.04±1.61	44.22±2.52	39.74±2.33
Extract1	17.98± 2.40	42.88± 2.57	39.14±1.93
Extract2	13.39± 2.06	51.27±2.70	35.34± 2.37

Supplementary Table S3. List of primers used for qRT-PCR analysis.

Accession number	Description (ITAG release 3.20)	Primer Code	Forward primer	Reverse primer
**Solyc01g073750.3**	*Glucan synthase-like protein*	01g073750-1	AGGAGGATTATGGAGTTGCTG	GCAACTTCCTCTTAACTTTACCAAA
**Solyc01g006370.3**	*Callose synthase*	01g006370-1	GAAGTGATTGCACGGGAAGC	CTGAAGCAGTCCACTGACCA
**Solyc11g005985.1**	*Callose synthase*	11g005980-1	AGTGGGAAGAACAGCTTCGG	CTTCTCCGTGCTTCGAGGTT
**Solyc11g005980.2**	Callose synthase 5	11g005980-2	CGTCGAGTCTTGCTTCTCGT	AGCTGAGCTCTGTCTGCTTG
**Solyc02g078230.2**	*CalS11-like*	02g078230-1	TCACCAGAGGTGATGCAGTTC	TCACGAACTCCCAGAATGGTG
**Solyc06g075570.1**	*Respiratory burst oxidase-like protein*	06g075570-2	AGAATGGGGCGGATGGAATC	ATCTCCCTCCTTCCCTGTGG
**Solyc11g072800.1**	*Respiratory burst oxidase-like protein*	11g072800-1	GCAAGAGGGTTCAAGAGCCT	GCTTTCCATTCCTATAGAGGGTCC
**Solyc02g064700.2**	*Protein kinase superfamily protein*	02g064700-2	TTGGGAAGGTTCTGGGGACT	ATGGTTTCCTGCTGTGTCGT
**Solyc06g005060.2**	*elongation factor 1-alpha*	06g005060-1	CTGTGCCGCATATCGCCTAA	TGACCAATGACCACGATGCT
**Solyc07g025390.2**	*Peptidyl-prolyl cis-trans isomerase-like 3*	07g025390-2	GAAAACGTGCTGCAACTCCC	ACTGTGTCTCGTTGCAGTGT
**Solyc05g055770.2**	*Basic leucine zipper and W2 domain-containing 2*	05g055770-1	CCAAGTCCAGTGCTACGAGG	TCACAAAGTTTTGCCTGCCC
